# Differential effects of dual and synergist-based insecticide-treated bed nets on pyrethroid resistance and *L995F/S* knockdown resistance mutation dynamics in *Anopheles gambiae* s.l. populations in south-western Burkina Faso

**DOI:** 10.1186/s13071-025-07190-3

**Published:** 2025-12-20

**Authors:** Madou Tapsoba, Wamdaogo Moussa Guelbeogo, Antoine Sanou, Soumanaba Zongo, Christelle Gogue, Siaka Debe, Kyra Arnett, Kelly Davis, Jenny Shannon, Peder Digre, Julia Mwesigwa, Kenzie Tynuv, Christen Fornadel, Sagnon N’Falé, Molly Robertson, Joseph D. Challenger, Gauthier Tougri, Adama Gansané, Hilary Ranson, Gnankiné Olivier, Joseph Wagman

**Affiliations:** 1 Centre National de Recherche et de Formation sur le paludisme, Ouagadougou, Burkina Faso; 2 Université Joseph KI-ZERBO, Ouagadougou, Burkina Faso; 3 Université Yembila-Abdoulaye TOGUYENI, Fada N’Gourma, Burkina Faso; 4 PATH, Washington, DC USA; 5 Innovative Vector Control Consortium, IVCC, Liverpool, UK; 6 Medical Research Council (MRC) Centre for Global Infectious Disease Analysis, Imperial College London, London, UK; 7 Global Fund to Fight AIDS, Tuberculosis and Malaria, Geneva, Switzerland; 8 Vector Biology, Liverpool School of Tropical Medicine, Liverpool, UK; 9 Université Nazi Boni, Bobo-Dioulasso, Burkina Faso

**Keywords:** *Anopheles gambiae* s.l., Pyrethroid resistance, *kdr* mutations, Dual-active-ingredient insecticidal bed nets, Burkina Faso

## Abstract

**Background:**

The introduction of next-generation insecticide-treated nets (ITNs) in Burkina Faso aims to mitigate pyrethroid resistance in malaria vectors. This study evaluated the impact of different ITN types on phenotypic resistance and *kdr* mutation frequencies in *Anopheles gambiae* sensus lacto (s.l.) populations across three health districts over 3 years.

**Methods:**

Annual mosquito collections were conducted in Banfora (where pyrethroid–chlorfenapyr nets had been distributed), Gaoua (pyrethroid-only ITNs) and Orodara (pyrethroid–piperonyl butoxide [PBO] ITNs). Two populations were analysed: adult females collected directly from the field and those reared from field-collected larvae. World Health Organization (WHO) susceptibility bioassays measured 24-h mortality after exposure to 1×, 5× and 10× concentrations of deltamethrin and alphacypermethrin, with and without pre-exposure to piperonyl butoxide. Frequencies of *kdr* mutations *L995F* and *L995S* were determined by polymerase chain reaction (PCR).

**Results:**

High-intensity resistance was observed in each study district, with mortality consistently below 45% and not reaching WHO thresholds even at 10× doses. PBO increased mortality, indicating metabolic resistance, but failed to restore full susceptibility. *L995F* predominated across all districts, years and mosquito populations.* L995S* remained low and variable. Pyr-only nets were associated with rising *L995F* frequencies and lower mortality in resistance assays. Pyrethroid (Pyr)–chlorfenapyr (CFR) nets improved mortality in resistance assays without increasing *kdr* prevalence. Pyr–PBO nets showed partial and inconsistent efficacy, with mosquitoes having mixed patterns in resistance assays. Similar patterns between field and laboratory-reared populations were observed.

**Conclusions:**

ITN type strongly influenced resistance dynamics. Dual-active ingredient (AI) nets, particularly Pyr–CFR, appear more effective in managing resistance. Integrated resistance management combining ITN rotation, routine monitoring and complementary interventions is essential to preserve vector control efficacy.

**Graphical abstract:**

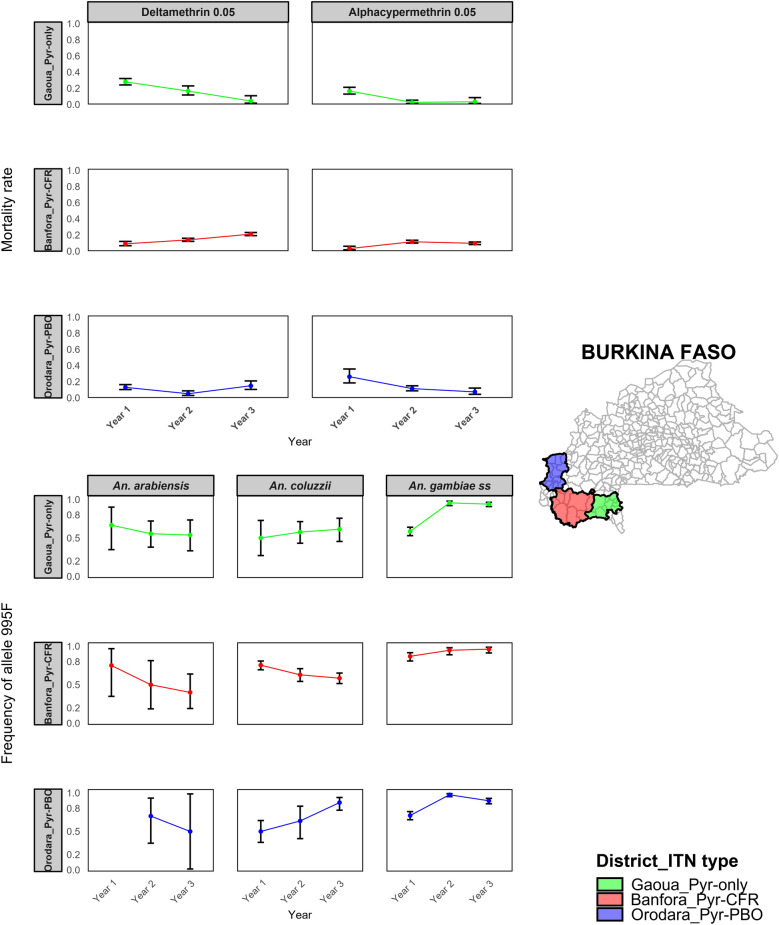

**Supplementary Information:**

The online version contains supplementary material available at 10.1186/s13071-025-07190-3.

## Background

In malaria-endemic countries, vector control strategies primarily rely on insecticide-treated bed nets (ITNs) and indoor residual spraying (IRS). Between 2000 and 2015, these interventions played a cornerstone role in preventing an estimated 663 million clinical cases in Sub-Saharan Africa [1]. Among these, approximately 68% (about 450 million cases) were attributed to the use of ITNs, 13% to IRS and 19% to artemisinin-based combination therapies, [1, 2]. During this period, more than 1 billion ITNs were distributed across the region, leading to a substantial rise in household ITN ownership from just 3% in 2000 to over 55% in 2015 [3]. Conversely, IRS coverage saw a marked decline after 2010, dropping from over 10% to below 2% of the population protected by 2019 [4, 5]. These trends reflect a strategic shift towards ITNs as the cornerstone of malaria vector control, largely due to their cost-effectiveness, operational simplicity and strong community uptake, facilitated through mass campaigns and routine distribution via antenatal care and child health services [6]. However, since 2015, these substantial gains have stagnated, largely owing to a declined efficacy resulting from the emergence and rapid spread of insecticide resistance (IR) among malaria vector populations across many African countries [7, 8].

In West Africa, IR was first reported in *Anopheles gambiae* complex populations in Côte d’Ivoire in 1993 [9]. Within a few years, resistance became widespread across most Sub-Saharan African countries [7, 8, 10, 11] including Burkina Faso, where pyrethroid resistance was first documented in 1999 [12]. Recent IR monitoring across Burkina Faso has confirmed that pyrethroid resistance is widespread and varies in intensity according to ecological zones and agricultural practices [13–16]. Between 2012 and 2018, studies reported alarmingly low mosquito mortality rates in World Health Organization (WHO) tube bioassays using 0.05% deltamethrin, reaching as low as 27% in Dano and 32% in Diébougou, indicating intense and operationally significant resistance [11, 17].

Resistance to insecticides in malaria vectors is mainly driven by two mechanisms: target-site mutations and metabolic detoxification by enzymes. The knockdown resistance (*kdr*) mechanism involves point mutations in the voltage-gated sodium channel (VGSC) gene, which reduce neuronal sensitivity to pyrethroids and dichlorodiphenyltrichloroethane (DDT) [18, 19]. The most common mutations of these *L995F* and *L995S* (formerly referred to as *L1014F* and *L1014S*, respectively) have been widely reported in *An. gambiae* sensus lacto (s.l.) populations across Africa, including Burkina Faso [14, 20, 21]. Furthermore, introgression between *An. gambiae* sensus stricto (s.s.) and *An. coluzzii* has facilitated the spread of *kdr* mutations [22]. Metabolic resistance, primarily mediated by cytochrome P450 monooxygenases, enhances insecticide detoxification. The overexpression of genes such as *CYP6P3*, *CYP6M2* and *CYP9K1* enables mosquitoes to metabolise pyrethroids more efficiently, thereby reducing their toxicity [23, 24]. Elevated P450 activity has been consistently observed in *An. gambiae* s.l. populations in Sub-Saharan Africa, including Burkina Faso [25] and correlates with reduced insecticide-induced mortality.

Recent studies have shown that the *L995F* mutation is closed to fixation in some populations and is often associated with the overexpression and gene amplification of detoxification enzymes such as *CYP6Z* and *CYP9K1* [26, 27]. The co-occurrence of *kdr* mutations and P450-mediated resistance can act synergistically to significantly compromise the effectiveness of ITNs [16, 28, 29].

The growing threat of IR compromises the efficacy of ITNs, necessitating innovative control strategies [30, 31]. Pyrethroid resistance in malaria vectors has been linked to reduced ITN-induced mortality and increased malaria transmission [32–34]. Evidence from modelling and experimental hut trials indicates that next-generation ITNs can help to restore efficacy in resistant areas [35, 36]. Among these tools, PermaNet^®^3.0, which combines deltamethrin (sides: 2.8 g/kg; roof: 4 g/kg) with the synergist piperonyl butoxide (PBO) (25 g/kg), and Interceptor^®^G2, combining alphacypermethrin (100 mg/m^2^) with chlorfenapyr (200 mg/m^2^), have shown promising results against resistant *An. gambiae* s.l. populations in Burkina Faso [37, 38]. These dual-active ingredient (dual-AI) ITNs offer enhanced efficacy by countering both metabolic and target-site resistance mechanisms.

During the 2019–2022 distribution campaign, the National Malaria Control Program (NMCP) of Burkina Faso piloted the deployment of Interceptor^®^G2 and PermaNet^®^3.0 in two health districts with documented high levels of pyrethroid resistance. Standard pyrethroid-only nets, which had been routinely distributed across the country since 2010, and these net types were introduced to evaluate their entomological and epidemiological impact in real-world conditions before consideration for nationwide scale-up. The objective of the pilot study was to assess whether these new-generation ITNs could overcome existing resistance mechanisms and restore control efficacy against malaria vectors. The study was conducted in collaboration with the NMCP of Burkina Faso, which, with support from donors and key stakeholders, was responsible for selecting the type of ITNs to be distributed and identifying the target deployment areas. The selection process was informed by malaria incidence data and trends in IR. In close coordination with local and international partners, the NMCP prioritized regions and districts with high levels of pyrethroid resistance to ensure that dual-AI ITNs were deployed in areas with the greatest need [39]. The study districts were assigned Interceptor^®^G2, PermaNet^®^3.0 and standard ITNs (Interceptor^®^) to evaluate their entomological and clinical impact before scaling the intervention nationwide.

While dual-AI ITNs such as Interceptor^®^G2 and PermaNet^®^3.0 have demonstrated enhanced efficacy against resistant mosquitoes in experimental settings, limited evidence exists on their long-term impact on resistance dynamics under operational conditions. In particular, it remains unclear whether sustained use of these nets can influence the trajectory of pyrethroid resistance and associated resistance mechanisms in wild type *An. gambiae* s.l. populations. Evaluating resistance trends over time following their deployment is, therefore, essential to assess their durability and guide future vector control strategies.

In the current paper, we explore the trend of pyrethroid resistance in *An. gambiae* s.l. over a 3-year period in south-western Burkina Faso, where dual-AI ITNs were distributed in 2019. Although no pre-distribution baseline was available, we used the first year post deployment as a reference point to monitor changes in phenotypic resistance and the frequency of *kdr* mutations (*L995F* and *L995S*). By tracking resistance indicators across multiple years, this study provides insights into the entomological impact of innovative ITNs and their potential role in mitigating the spread of resistance in malaria vectors.

## Methods

### Study area

**Fig. 1:**
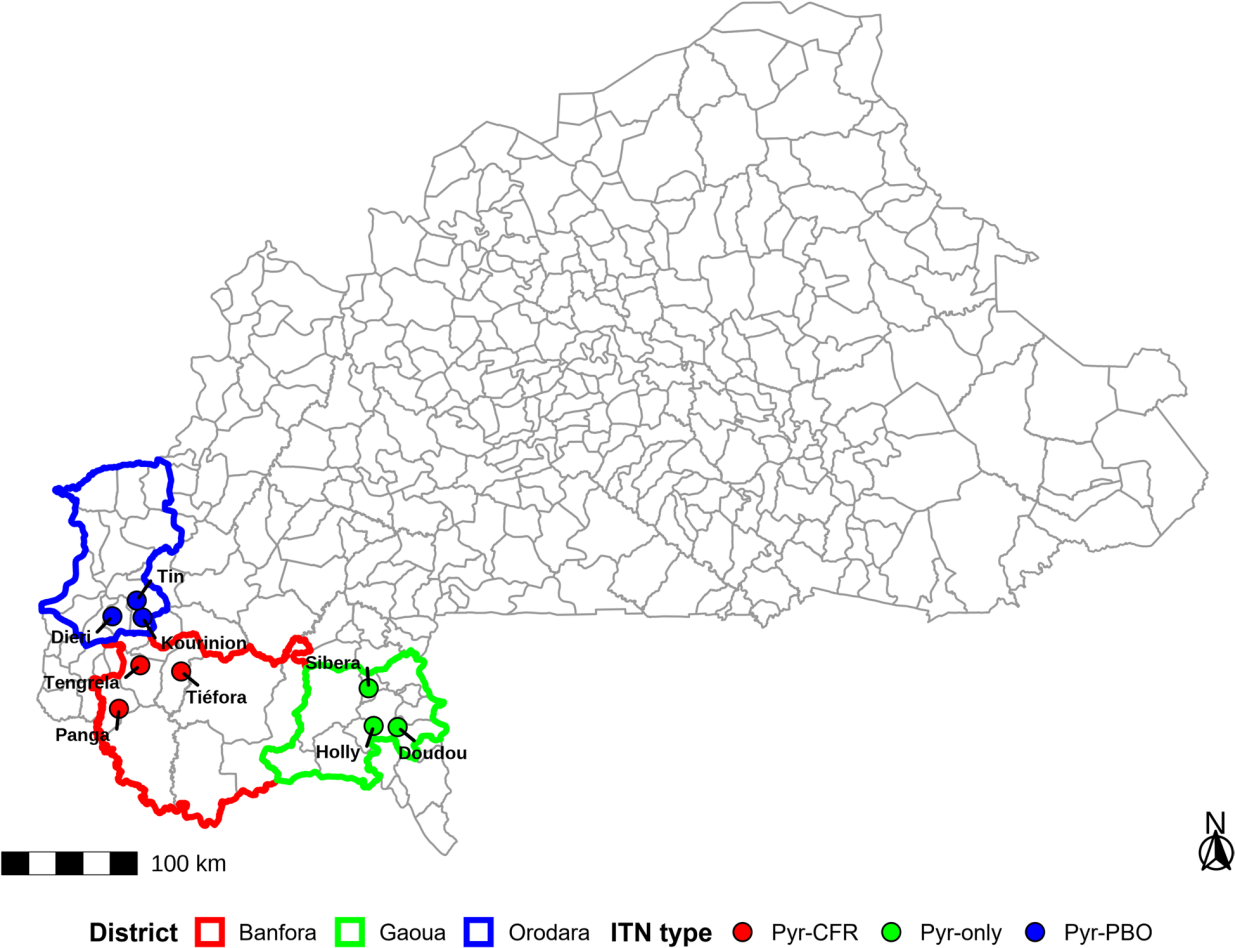
Map of the study health districts in south-western Burkina Faso. The map showing the location of the three study districts, Banfora, Gaoua and Orodara, within the national borders of Burkina Faso. All provincial boundaries are displayed in light grey. The three study districts are outlined in bold colour: Banfora (red), Gaoua (green) and Orodara (blue). The surveyed villages are represented by coloured dots, with each colour corresponding to the type of insecticide-treated net (ITN) deployed: red for Pyr–CFR, green for Pyr-only and blue for Pyr–PBO. The positions of the villages were recorded using handheld Global Positioning System (GPS) devices during field activities. A north arrow and a metric scale bar are included for geographic reference. *Pyr–CFR* pyrethroid + chlorfenapyr, *Pyr-only* pyrethroid-only net, *Pyr–PBO* pyrethroid + piperonyl butoxide. This figure was generated using spatial shapefiles from administrative datasets and field-collected geographic coordinates. It is intended to accompany the manuscript text and illustrate the spatial distribution of intervention arms

The study was conducted in three health districts in south-western Burkina Faso: Orodara, Banfora and Gaoua. In each district, three villages were selected for the study: Tengrela, Tiéfora and Panga in Banfora; Diéri, Kourinion and Tin in Orodara; and Sibera, Holly and Doudou in Gaoua, as shown in the map (Fig. 1). Orodara and Banfora, where high levels of pyrethroids resistance have been documented, were selected as intervention sites to evaluate respectively the effect of PermaNet^®^3.0 (Vestergaard) and Interceptor^®^G2 (BASF) on pyrethroid resistance in local *An. gambiae* s.l. populations. Gaoua, which shares similar characteristics with Banfora and Orodara in terms of malaria prevalence, transmission intensity and *An. gambiae* s.l. insecticide resistance served as the comparison district and received pyrethroid-only ITNs (Interceptor^®^, manufactured by BASF).

Importantly, all three districts exhibit comparable agricultural practices, particularly the widespread cultivation of cotton and other crops that rely heavily on pesticide use including pyrethroids and organophosphates for pest management. The intensive and often unregulated application of these agricultural insecticides is considered a key environmental driver of insecticide resistance in malaria vectors. Studies have demonstrated that pesticide residues from agricultural activities can contaminate mosquito breeding sites, exerting selection pressure that contributes to the development of resistance mechanisms such as *kdr* mutations in *An. gambiae* s.l. populations [17, 40–42].

The study area is characterised by a humid savannah climate with a rainy season from May to October and a dry season from November to April. Annual rainfall ranges from 900 to 1200 mm, with an average annual temperature of approximately 27 °C. The average relative humidity ranged around 25% during the dry season to 85% during the rainy season.

### *Anopheles gambiae* s.l. larvae collection

From August 2019 to March 2022, annual larval collections were conducted by standard dipping from multiple breeding sites across the three sentinel villages in each health district. Larvae from sentinel sites in each district were pooled to constitute a district-representative sample. They were then transported to the insectary at the Centre National de Recherche et de Formation sur le Paludisme (CNRFP) in Banfora, where they were reared to adulthood under controlled conditions (temperature 27 ± 2 °C, relative humidity 70 ± 10%).

### Pyrethroid insecticide susceptibility and resistance intensity bioassays

Before conducting tests with the insecticide-impregnated papers, the reference strain *An. gambiae* "Kisumu" maintained in the laboratory, was used to validate the quality of the impregnated papers. Then 20–25 sugar-fed *An. gambiae* s.l. females aged 3–5 days were exposed to deltamethrin, alphacypermethrin and PBO-treated papers manufactured by the WHO/Vector Control Research Unit at University Sains Malaysia (WHO/VCRU). The insecticide bioassays were conducted according to WHO guidelines for tube bioassay [43].

After an initial 1-h observation period to assess the fitness and activity of the test mosquitoes, they were subsequently exposed to insecticide-treated papers for 1 h under standardized laboratory conditions (temperature 27 ± 2 °C, relative humidity 70 ± 10%). For each test, four replicates of 20–25 mosquitoes each (approximately 100 mosquitoes in total) were conducted per selected health district.

Two pyrethroid insecticides, deltamethrin and alphacypermethrin, were used for susceptibility assays in each health district. To monitor pyrethroid resistance intensity, papers treated with deltamethrin at 0.05% and alphacypermethrin at 0.05%, which are considered to be the diagnostic concentrations, and five then ten times these diagnostic doses were used. For assessing the implication of metabolic resistance, bioassays were conducted by pre-exposing 100 sugar-fed female *An. gambiae* s.l., aged 3–5 days to PBO-4%-treated paper then to diagnostic concentrations of deltamethrin or alphacypermethrin for an additional hour. Furthermore, two negative control replicates (approximately 50 mosquitoes) per test were performed using filter papers treated with silicone oil. After exposure, mosquitoes were held under laboratory conditions for 24 h (temperature 27 ± 2 °C, relative humidity 70 ± 10%) to assess mortality.

### Adult mosquito collection and laboratory processing

Concomitantly to larval collection, adult mosquitoes were also collected using Human Landing Catches (HLC) and Center for Disease Control Light Trap (CDC-LT) in the nine villages of the study. Nightly mosquito collections were performed once a week; from 7 pm to 6 am using HLC and from 8 pm to 6 am with the CDC-LT. After collection, mosquitoes were transferred to the entomological laboratory of each health district for morphological identification of species or species complex using the dichotomous morphological keys [44] sorted according to the physiological status as fed, unfed, gravid and semi-gravid. All *Anopheles* mosquitoes were collected and preserved in pre-labelled 1.5 ml Eppendorf tubes containing silica gel at room temperature; however, only *Anopheles gambiae* s.l. specimens were used for molecular analysis.

### Molecular analysis

Two subsets of *An. gambiae* s.l. adults were selected at the district level after pooling collections across the three sentinel villages for molecular analysis. The first subset targeted approximately 300 wild-caught adult females per district per year. The second subset target approximately 75 adult mosquitoes per district per year from the negative controls groups of the bioassays; these mosquitoes originated from field-collected larvae reared to adulthood under insectary and were exposed to untreated control papers. For both subsets, molecular processing was conducted in two stages: (i) species identification within the *An. gambiae* complex; and (ii) *kdr* genotyping (*L995F* and *L995S*) performed only on specimens with successful species identification.

For these molecular analysis, genomic DNA was extracted from individual *An. gambiae* s.l. using the DNAzol isolation kit (Invitrogen by Thermo Fisher Scientific), re-suspended in 100 μl sterile water and stored at −20 °C.

### *Anopheles gambiae* complex members’ identification

Extracted genomic DNA was used for *An. gambiae* complex identification at species level by standard polymerase chain reaction (PCR) using method of Fanello and others [45]. The amplified DNA product was electrophoresed in Tris–Borate–ethylenediaminetetraacetic acid (EDTA) buffer on 2% agarose gel and visualized under ultraviolet transilluminator after staining with Redsafe.

### *kdr* target-site mutation genotyping

*kdr* target-site mutation genotyping was performed using the TaqMan PCR method as described in ref. [46] to investigate the *kdr L995F* and *L995S* mutation presence in the VGSC gene. Assays were separately performed for each *kdr* mutation (*L995F* and *L995S*) using 10 µL PCR reaction mix for each sample. The mix contained 5 µL Luna Universal qPCR Master Mix, 0.9 pmol/μL *kdr*_Forward (5′-CATTTTTCTTGGCCACTGTAGTGAT-3′) and *kdr*_Reverse (5′-CGATCTTGGTCCATGTTAATTTGCA-3′) primers, 0.2 pmol/µL probes labelled with HEX fluorophore (WT, 5′-CTTACGACTAAATTTC-3′) for wild type allele detection and FAM fluorophores (5′-ACGACAAAATTTC-3′ or 5′-ACGACTGAATTTC-3′, respectively, for *kdr L995F* and *L995S* detection). The reaction mixture also included 1 pmol/µL genomic DNA extract from individual *Anopheles gambiae* s.l. head-thorax and 1.8 µL sterile water. PCR was run on the Aligent Mx Pro 3005P machine using TaqMan technology.

### Data analysis

Data were recorded in Microsoft Excel and analysed using the free R statistical software version 4.4.1. [47]. The 24-h post-exposure mortality rate was estimated as the proportion of dead over total exposed individuals by health district and results interpretation followed the WHO insecticide resistance monitoring guidelines [43]. *An. gambiae* s.l. population was considered resistant to pyrethroid insecticide when the 24-h post-exposure mortality rate at the diagnostic dose was less than 90%. Susceptibility to pyrethroid insecticides was considered as moderate intensity if the mortality rate exceeded 98% at five times the diagnostic concentration (DC). When the mortality rate was less than 98% at this concentration, moderate-to-high intensity resistance was suspected. If mortality was greater than 98% at ten times the DC, the resistance was classified as moderate intensity. If it was still below 98% at this concentration, the resistance was classified as high intensity. To evaluate the variation in 24-h mosquito mortality across different years within each health district, a logistic regression model (GLM) with a logit link function and binomial distribution was employed. The model incorporated year, ITN type and their interaction as covariates to predict the probability of mosquito mortality. All the GLMs were performed using the lme4 R package.

Each year of the study, the allelic frequencies of the *kdr L995F* and* L995S* mutations were estimated in *An. gambiae* s.l. populations sampled within each health district, including adult mosquitoes collected directly from the field and adults reared in the laboratory from larvae collected in the field. As the PCR assays for *L995F* and *L995S* were performed separately, the genotyping data from both assays were first combined for each individual mosquito to accurately determine its genotype before calculating allelic frequencies (Additional File 1: Text S1). The comparison of *kdr L995F* mutation frequencies between years and mosquitoes populations in each health district was then performed using a chi-squared test.

## Results

### *Anopheles gambiae* complex species composition

Of the 3375 specimens processed under our two-stage molecular workflow, species identification within the *An. gambiae* complex followed by *kdr* (*L995F/L995S*) genotyping only for identification-positive specimens, 1589 female *An. gambiae* s.l. achieved successful outcomes at both steps across the three health districts over the 3 years. These fully genotyped counts arise from district-level, target-based subsampling after pooling the three sentinel villages (~300 wild-caught adult females and ~75 adults per district-year from untreated control groups) and therefore reflect successful outcomes rather than totals collected; they comprise 1125 (70.8%) wild-caught adult females and 464 (29.2%) adults reared from field-collected larvae. Among the field-collected adults, the distribution across the three districts was relatively balanced: 376 (33.4%) from Gaoua, 372 (33.1%) from Banfora and 377 (33.5%) from Orodara (Table 1). Similarly, among the adults reared from wild-caught larvae, 162 (34.9%) originated from Gaoua, 147 (31.7%) from Banfora and 155 (33.4%) from Orodara (Table 1). Table 1 reports only specimens with successful species identification and *kdr* genotyping; samples failing at either step were excluded.

**Table 1:** Species composition of adult wild *Anopheles gambiae* s.l. populations collected directly from the wild type strain compared with those from F0 adults obtained in the laboratory from field-collected larvae, by health district and study year

Collection year	*Anopheles* species	Banfora (Pyr–CFR area)		Gaoua (Pyr-only area)		Orodara (Pyr–PBO area)	
		Wild type	F0	Wild type	F0	Wild type	F0
Year 1	*An. gambiae* s.s. (*n*/*N*) P (%) [95%CI]	73/160 45.6 [37.9–53.3]	11/46 23.9 [11.6–36.2]	148/159 93.1 [89.1–97.0]	15/20 75.0 [56.0–94.0]	129/152 84.9 [79.2–90.6]	23/26 88.5 [76.2–100]
	*An. coluzzii* (*n*/*N*) P (%) [95%CI]	86/160 53.8 [46.0–61.5]	32/46 69.6 [56.3–82.9]	10/159 6.3 [2.5–10.1]	0/20 –	23/152 15.1 [9.4–20.8]	3/26 11.5 [0.0–23.8]
	*An. arabiensis* (*n*/*N*) P (%) [95%CI]	1/160 0.6 [0.0–1.8]	3/46 6.5 [0.0–13.7]	1/159 0.6 [0.0–1.9]	5/20 25.0 [6.0–44.0]	0/152 –	0/26 –
Year 2	*An. gambiae* s.s. (*n*/*N*) P (%) [95%CI]	33/81 40.7 [30.0–51.4]	24/51 47.1 [33.4–60.8]	72/84 85.7 [78.2–93.2]	42/74 56.8 [45.5–68.0]	77/89 86.5 [79.4–93.6]	71/75 94.7 [78.2–93.2]
	*An. coluzzii* (*n*/*N*) P (%) [95%CI]	48/81 59.3 [48.6–69.9]	22/51 43.1 [29.5–56.7]	10/84 11.9 [5.0–18.8]	16/74 21.6 [12.2–31.0]	11/89 12.4 [5.5–19.2]	0/75 –
	*An. arabiensis* (*n*/*N*) P (%) [95%CI]	0/81 –	5/51 9.8 [1.6–18.0]	2/84 2.4 [.0–5.6]	16/74 21.6 [12.2–31.0]	1/89 1.1 [0.0–3.3]	4/75 5.3 [0.2–10.4]
Year 3	*An. gambiae* s.s. (*n*/*N*) P (%) [95%CI]	52/131 39.7 [31.3–48.1]	13/50 26.0 [13.8–38.1]	120/132 90.2 [85.2–95.3]	46/68 67.6 [56.5–78.8]	104/136 76.5 [69.3–83.6]	49/54 90.7 [83.0–98.5]
	*An. coluzzii* (*n*/*N*) P (%) [95%CI]	75/131 57.3 [48.8–65.7]	31/50 62.0 [48.5–75.4]	7/132 5.3 [1.5–9.1]	15/68 22.1 [12.2–31.9]	32/136 23.5 [16.4–30.7]	4/54 7.4 [0.4–14.4]
	*An. arabiensis* (*n*/*N*) P (%) [95%CI]	4/131 3.1 [0.1–6.0]	06/50 12.0 [3.0–21.0]	6/132 4.5 [1.0–8.0]	7/68 10.3 [3.1–17.5]	0/136 –	1/54 1.9 [0.0–5.4]

Proportions (P, %) and their 95% confidence intervals (CI) are presented for each molecularly identified species (*An. gambiae* s.s., *An. coluzzii*, *An. arabiensis*) within the *An. gambiae* complex. Wild type strain refers to adult mosquitoes collected directly in the field using human landing catches and CDC light traps. F0 strain refers to adult mosquitoes reared in the laboratory from larvae collected in the field. *n*/*N* is the number of individuals identified per species (*n*) out of the total analysed (*N*). Pyr-only area indicates the district where Interceptor^®^ ITNs were deployed (pyrethroid-only nets). Pyr–CFR area indicates the district where Interceptor^®^G2 ITNs were deployed (pyrethroid + chlorfenapyr nets). Pyr–PBO area indicates the district where PermaNet^®^3.0 ITNs were deployed (pyrethroid + PBO nets). – indicates that no individuals of that species were detected or the proportion could not be calculated

Counts in this table reflect only specimens with successful species identification and *kdr* genotyping; samples failing at either step were excluded. Variation across districts/years arises from field availability, operational constraints and laboratory QC; molecular subsampling targets were set at the district level after pooling villages within each district

Species composition differed by district and collection method: in Gaoua and Orodara, *An. gambiae* s.s. was the most abundant species within the *An. gambiae* complex in both field-collected and adults reared from wild-caught larval mosquito populations; whereas in Banfora, *An. gambiae* s.s. and *An. coluzzii* were found in nearly equal proportions across both collection methods. The proportions of *Anopheles gambiae* s.s., *An. coluzzii*, and *An. arabiensis* by district, year and collection methods are presented in Table 1.

Significant differences in species composition between field-collected adult mosquitoes (sampled continuously throughout the entire year) and adults reared from wild-caught larval mosquitoes derived from field-collected larvae (collected only during the rainy season) were observed in most cases. These differences were supported by *P*-values below 0.05 for the majority of comparisons, particularly in Banfora (year 1: *P* = 0.003; year 2: *P* = 0.006; year 3: *P* = 0.03), Gaoua (year 1: *P* < 0.0001; year 2: *P* < 0.0001; year 3: *P* < 0.0001) and Orodara (year 2: *P* = 0.0004). Conversely, no significant difference was observed in Orodara in year 1 (*P* = 0.7) and year 3 (*P* = 0. 06).

### Pyrethroid resistance trend in *An. gambiae* s.l. populations

**Fig. 2:**
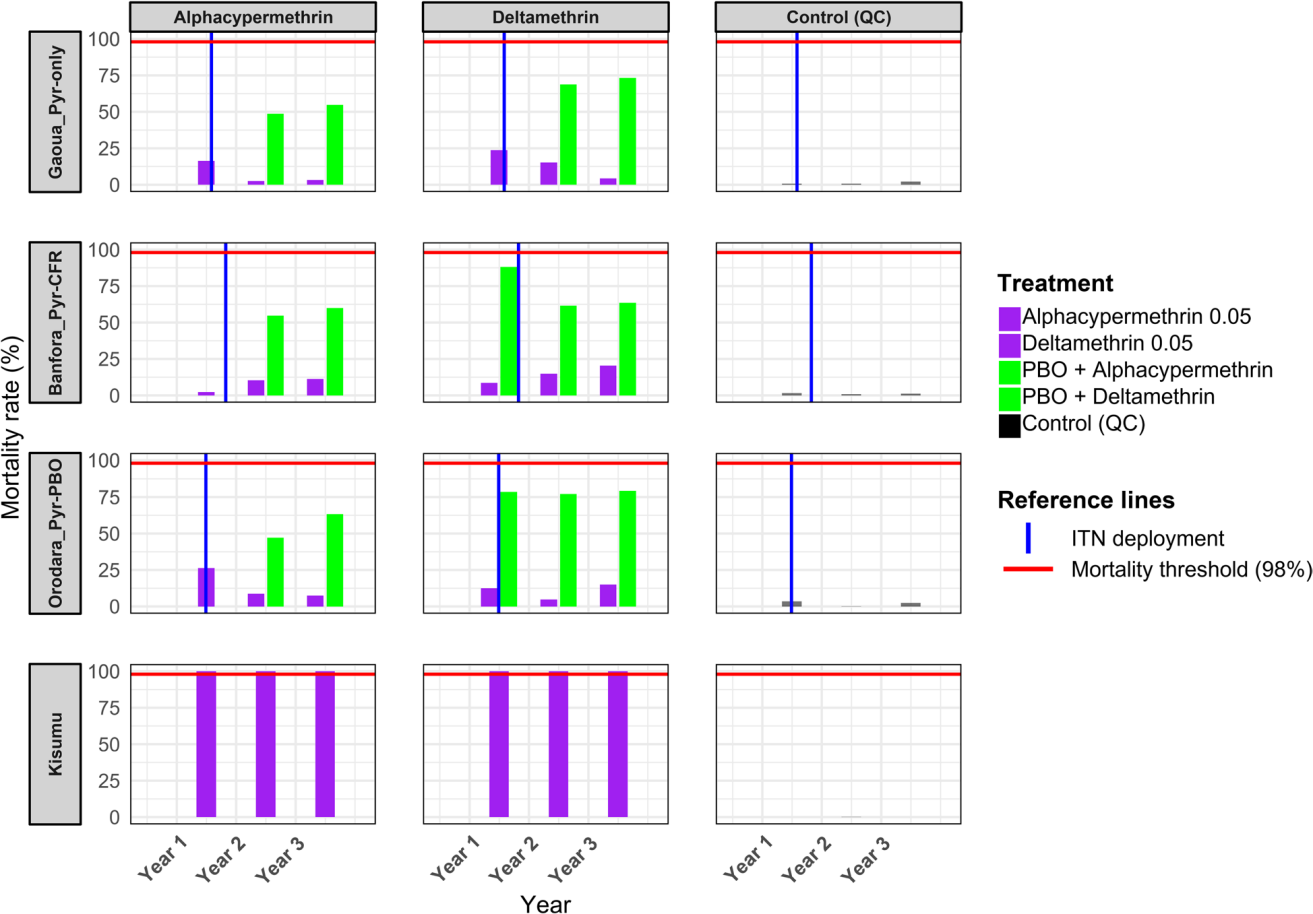
The 24-h mortality rates (%) of *Anopheles gambiae* sensu lato populations following exposure to pyrethroid insecticides diagnostic concentration with (PBO_Alphacypermethrin or PBO_Deltamethrin) or without (Alphacypermethrin_0.05 or Deltamethrin_0.05) pre-exposure to piperonyl butoxide (PBO), across study years (2019–2021) and health districts. Each point represents the mortality observed in a single susceptibility test. Health districts are grouped according to the type of insecticide-treated net (ITN) deployed during the intervention: Banfora received Interceptor^®^G2 nets (Pyr–CFR), Gaoua received Interceptor^®^ nets (Pyr-only) and Orodara received PermaNet^®^3.0 nets (Pyr + PBO). The Kisumu strain represents a susceptible laboratory colony used as a reference. The vertical blue dashed line indicates the specific date of ITN deployment in each district, while the horizontal red dotted line represents the 90% mortality threshold recommended by WHO to indicate full susceptibility. *PBO* piperonyl butoxide, *ITNs* insecticide-treated nets

**Fig. 3:**
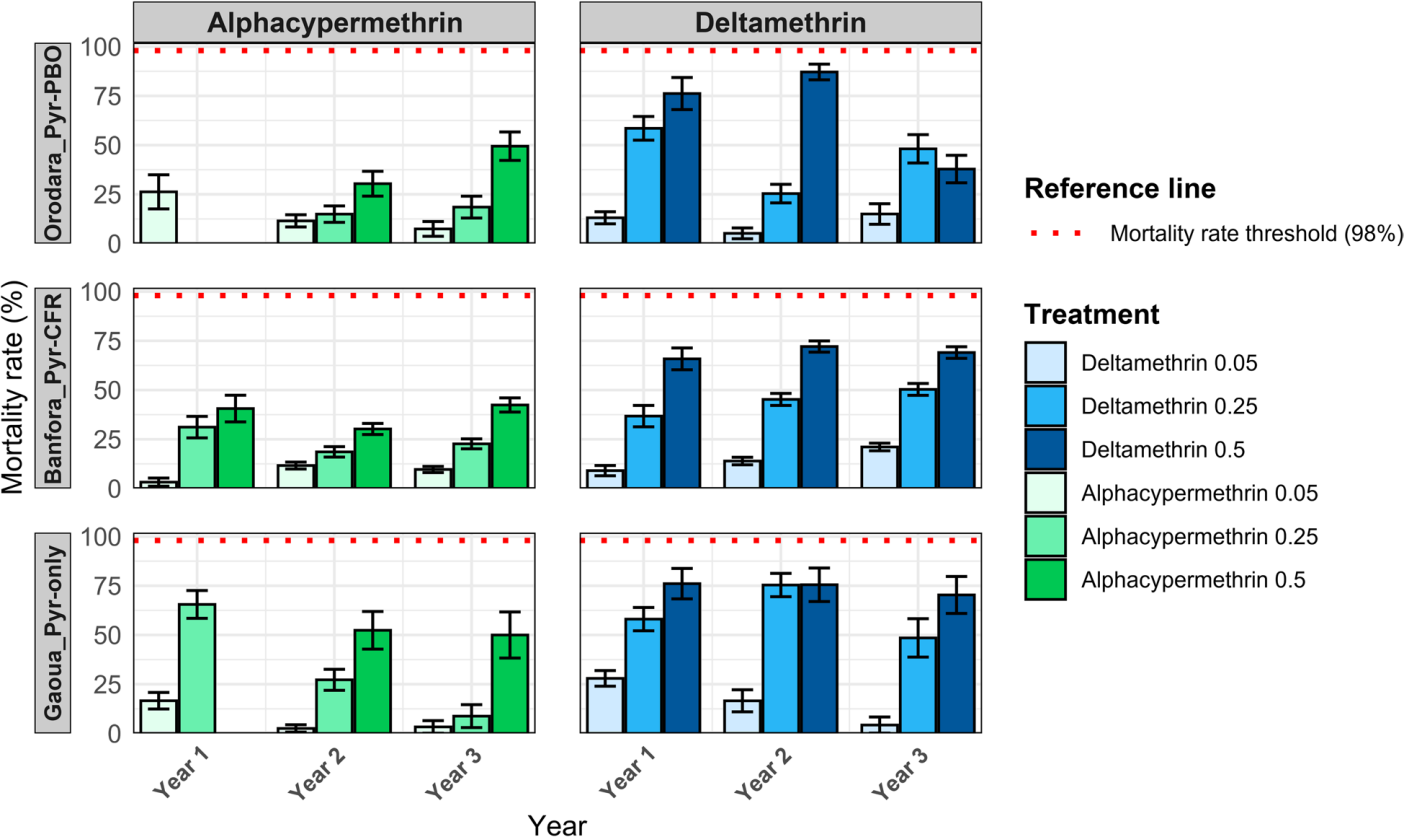
The 24-h mortality rates (%) of *Anopheles gambiae* sensu lato populations following exposure to increasing diagnostic concentrations (1×, 5× and 10×) of alphacypermethrin and deltamethrin, by year of mosquito collection (2019–2021) and health district. Bars represent the mean mortality observed in each treatment, with error bars indicating the standard error. Health districts are grouped according to the type of insecticide-treated bed net (ITN) deployed during the intervention: Banfora (Interceptor^®^G2, Pyr–CFR), Gaoua (Interceptor®, Pyr-only) and Orodara (PermaNet^®^3.0, Pyr + PBO). The horizontal red dotted line represents the WHO-defined 98% mortality threshold indicating full susceptibility. *ITNs* insecticide-treated nets

Throughout the 3 years of the study, the mortality rate of mosquitoes exposed to filter papers impregnated with silicone oil remained below 5%. In contrast, the mortality rate of the laboratory reference strain *An. gambiae* Kisumu exposed to insecticide-impregnated papers was consistently around 100% across all the three study districts (Fig. 2). Over the 3-year study period, the 24-h mortality rate of *An. gambiae* s.l. following exposure to pyrethroid insecticides diagnostic concentration remained consistently below 45% across all the study areas (Fig. 2). Exposure to five and ten times the diagnostic concentration of deltamethrin and alphacypermethrin resulted in mortality rate below 98% across all the three study areas throughout the 3 years of the study (Fig. 3).

**Fig. 4:**
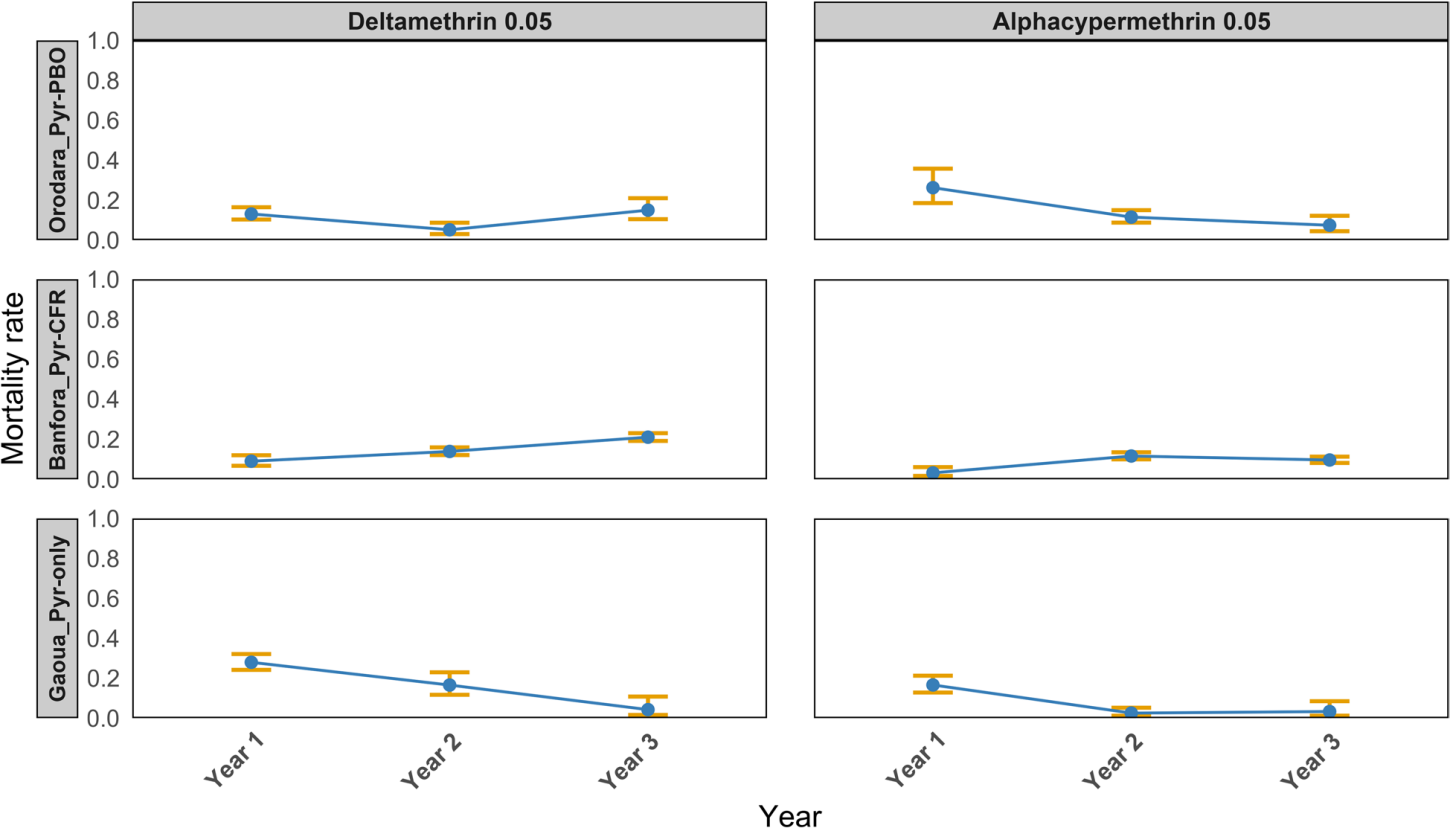
Temporal trends in the susceptibility of *Anopheles gambiae* sensu lato populations to diagnostic concentration of pyrethroid insecticides (Deltamethrin_0.05 and Alphacypermethrin_0.05) across the three study health districts, Banfora, Gaoua and Orodara from 2019 to 2021. Each point represents the mean 24-h mortality rate (expressed as a proportion) observed in WHO susceptibility bioassays for a given treatment and district. Vertical error bars indicate the standard error of the mean. The *y* axis is displayed on a proportional scale (0–1). The three districts correspond to different types of insecticide-treated nets (ITNs) deployed during the study. *Pyr–CFR* pyrethroid–chlorfenapyr ITN (Interceptor^®^G2, Banfora), *Pyr-only* pyrethroid-only ITN (Interceptor^®^, Gaoua), *Pyr–PBO* pyrethroid–PBO ITN (PermaNet^®^3.0, Orodara). Susceptibility was assessed according to WHO tube bioassay protocols

In the Gaoua health district, where Pyr-only bed nets were deployed, there was a progressive decline in mosquito mortality following exposure to 0.05% deltamethrin over time. In 2019, the mortality rate was 27.9% (95% confidence interval [CI] 24.1–32.0), which significantly decreased to 16.6% (95% CI 11.3–23.0, *P* < 0.01) in 2020 and further dropped to 3.3% (95% CI 1.2–10.5, *P* < 0.01) in 2021 (Fig. 4). A similar trend was observed with 0.05% alphacypermethrin, where the mortality rate fell from 16.6% (95% CI 12.6–21.3) in 2019 to 2.5% (95% CI 1.0–5.2, *P* < 0.0001) in 2020 and remained low at 3.3% (95% CI 0.9–8.2, *P* < 0.0001) in 2021.

In the Banfora health district, where dual active ingredient net (Pyr–CFR bed nets) were deployed, the mortality rate of mosquitoes after exposure to 0.05% deltamethrin increased from 9.1% (95% CI 6.6–12.1) in 2019 to 13.9% (95% CI 12.1–16.0, *P* < 0.01) in 2020 and further rose to 21.1% (95% CI 19.1–23.1, *P* < 0.01) in 2021 (Fig. 4). Similarly, for 0.05% alphacypermethrin, the mortality rate increases significantly from 3.2% (95% CI 1.5–6.1) in 2019 to 11.7% (95% CI 9.9–13.5, *P* < 0.0001) in 2020, before slightly decreasing to 9.7% (95% CI 8.1–11.4, *P* < 0.0001) in 2021.

In the Orodara health district, where Pyr–PBO bed nets were used, the mortality rate of *An. gambiae* s.l. populations following exposure to 0.05% deltamethrin decreased significantly from 13.1% (95% CI 10.2–16.5) in 2019 to 5.2% (95% CI 2.8–8.7, *P* = 0.03) in 2020, before increasing to 15.0% (95% CI 10.1–21.1, *P* = 0.03) in 2021 (Fig. 4). For 0.05% alphacypermethrin, the mortality rate significantly declined from 26.3% (95% CI 17.9–36.1) in 2019 to 11.5% (95% CI 8.6–15.0, *P* = 0.04) in 2020 and further decreased to 7.4% (95% CI 4.1–12.2, *P* = 0.6) in 2021. The trends in mosquito mortality rates following exposure to 0.25% alphacypermethrin and 0.25% deltamethrin are presented in Additional File 2: Supplementary Fig. S1, while the trends following exposure to 0.5% alphacypermethrin and 0.5% deltamethrin are shown in Additional File 2: Supplementary Fig. S2.

### Metabolic resistance investigation in *An. gambiae* s.l.

Pre-exposure to the synergist PBO 4% followed by a pyrethroid insecticide significantly increased mortality rates in all study areas but remained below the 98% threshold (Fig. 2).

In the Gaoua health district, where Pyr-only bed nets were deployed, pre-exposure to the synergist PBO 4% followed by 0.05% deltamethrin significantly increased the mortality rate of *An. gambiae* s.l. populations, reaching 68.5% (95% CI 58.5–77.8, *P* < 0.0001) in 2020 and 73.3% (95% CI 63.5–81.6, *P* < 0.0001) in 2021 (Fig. 2). Similarly, PBO 4% pre-exposure followed by 0.05% alphacypermethrin resulted in a mortality rate of 48.7% (95% CI 37.0–60.4, *P* < 0.0001) in 2020 and 54.7% (95% CI 44.2–65.0, *P* < 0.0001) in 2021 (Fig. 2).

In the Banfora health district, where Pyr–CFR bed nets were in used, pre-exposure to PBO 4% followed by 0.05% deltamethrin significantly increased the mortality rate to 61.9% (95% CI 57.5–66.1, *P* < 0.001) in 2020 and 70.0% (95% CI 67.3–72.6, *P* < 0.001) in 2021(Fig. 2). Pre-exposure to PBO 4% followed by 0.05% alphacypermethrin also resulted in a significant increase in mortality rate, reaching 54.9% (95% CI 51.2–58.6, *P* < 0.001) in 2020 and 61.2% (95% CI 58.–64.4, *P* < 0.001) in 2020 (Fig. 2).

In the Orodara health district, where Pyr–PBO beds nets were deployed, pre-exposure to PBO 4% followed by 0.05% deltamethrin significantly increased mortality rates, reaching 75.6% (95% CI 68.5–81.8, *P* < 0.001) in 2020 and 79.3% (95% CI 72.7–84.8, *P* < 0.001) in 2021 (Fig. 2). Similarly, PBO pre-exposure followed by 0.05% alphacypermethrin resulted in significant increases in mortality, with rates of 46.2% (95% CI 39.2–53.4, *P* < 0.0001) in 2020 and 63.4% (95% CI 55.7–70.6, *P* < 0.001) in 2021 (Fig. 2).

### Trends in allelic frequencies of *kdr L995F* and *L995S* mutations

The *kdr L995F* and *L995S* mutations were observed at varying frequencies across the study districts and mosquito populations, both in adults collected directly from the field and in adults reared from wild-caught larvae, as well as among members of the *An. gambiae* complex (Additional File 3: Supplementary Table S1). Overall, the frequency of the *L995F* mutation was significantly higher than that of the *L995S* mutation across all districts, mosquito populations and study years (*t* = 26.825, *df* = 17, *P* < 0.0001). There was no significant difference in *L995F* allele frequency between wild adult mosquitoes collected in the field and adults reared from wild-caught larvae (*t* = −0.874, *df* = 12.57, *P* = 0.3985). Conversely, the frequency of the *L995S* allele was statistically higher in wild adult mosquitoes collected in the field compared with adults reared from wild-caught larvae (*t* = 2.378, *df* = 12.36, *P* = 0.0343). It should be noted that in some cases, particularly for *An. arabiensis* and *An. coluzzii*, the number of mosquitoes analysed was below 30 individuals. These small sample sizes may limit the robustness of allele frequency estimates and could lead to an overestimation of the results. Therefore, the frequencies reported for these species should be interpreted with caution.

**Fig. 5:**
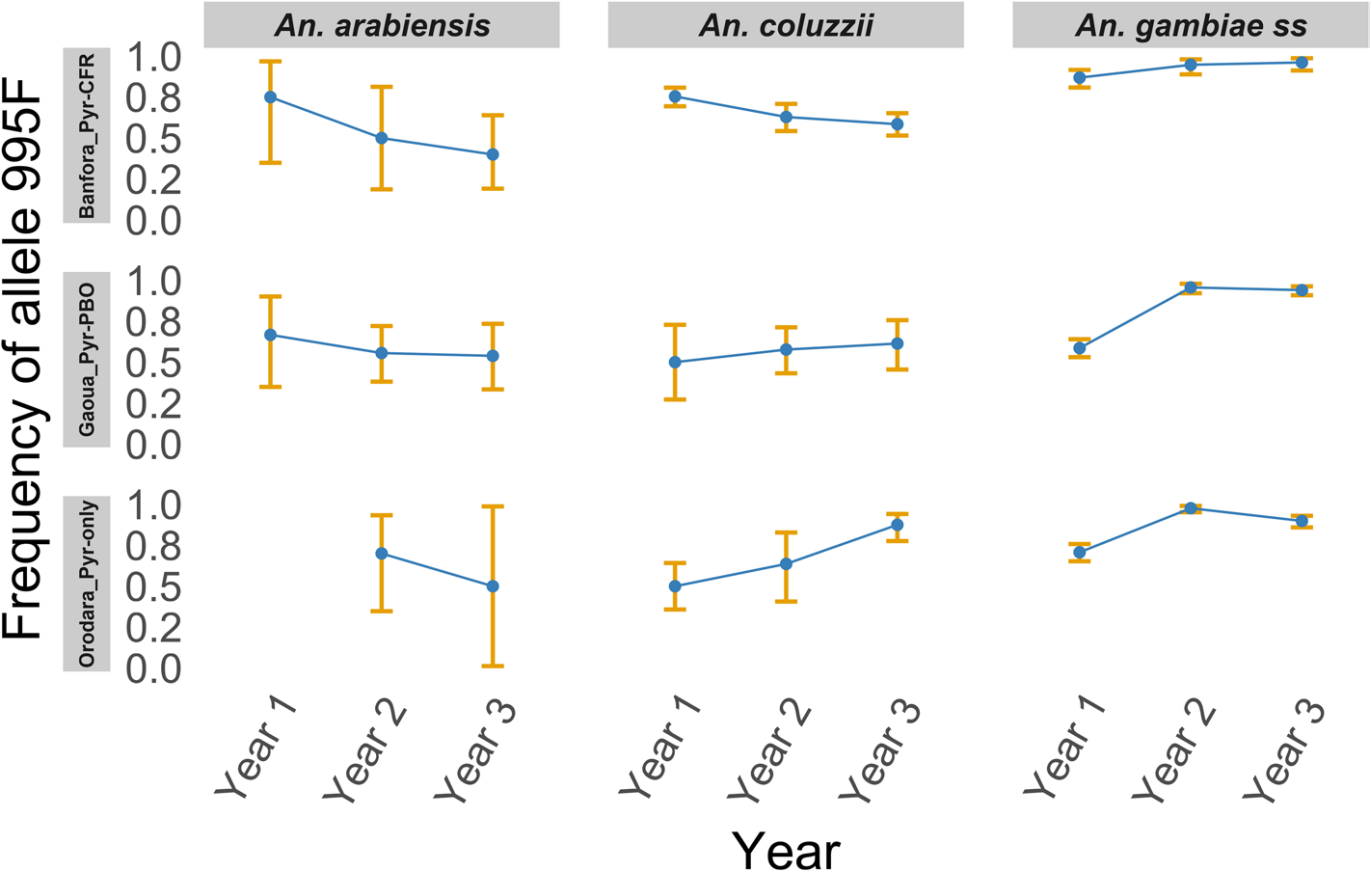
Temporal trends in the frequency of the *kdr* *L995F* mutation in three molecular forms of *Anopheles gambiae* s.l. (*An. gambiae*, *An. coluzzii* and *An. arabiensis*) over 3 consecutive years in Banfora (with Interceptor^®^G2 deployment), Gaoua (with Interceptor® deployment) and Orodara (with PermaNet^®^3.0 deployment) health districts. The *y* axis shows the estimated *L995F* allele frequency, and panels are labelled by district and ITN type deployed. Points represent the mean allele frequencies per year, with vertical orange bars indicating 95% confidence intervals. This analysis highlights spatial and temporal variations in the prevalence of the *L995F* resistance allele among vector populations exposed to different ITN types

The annual frequencies of the *kdr L995F* and *L995S* mutations were estimated in *An. gambiae* s.l. populations, combining wild adult mosquitoes collected directly from the field and adults reared from wild-caught larvae, across three health districts from 2019 to 2021. In Banfora, the frequency of the *L995F* mutation remained relatively stable over the study period. It was 0.80 (95% CI 0.75–0.86) in 2019, slightly decreased to 0.76 (95% CI 0.69–0.83) in 2020 (2019 versus 2020: *χ*^2^ = 0.532, *P* = 0.466), and further to 0.71 (95% CI 0.64–0.78) in 2021 (2019 versus 2021: *χ*^2^ = 3.874, *P* = 0.049). However, these changes were not strongly significant and may reflect relatively consistent allele frequencies across the years. In Gaoua, a marked increase in *L995F* frequency was observed: 0.58 (95% CI 0.51–0.66) in 2019, 0.85 (95% CI 0.79–0.90) in 2020 (2019 versus 2020: *χ*^2^ = 27.078, *P* < 0.0001) and 0.88 (95% CI 0.84–0.93) in 2021 (2019 versus 2021: *χ*^2^ = 46.416, *P* < 0.0001). In Orodara, the frequency increased from 0.68 (95% CI 0.61–0.75) in 2019 to 0.95 (95% CI 0.91–0.98) in 2020 (2019 versus 2020: *χ*^2^ = 37.492, *P* < 0.0001). A slight decline was noted in 2021 with the *L995F* allele frequency of 0.89 (95% CI 0.85–0.94; 2020 versus 2021: *χ*^2^ = 24.194, *P* < 0.0001). Figure 5 shows these temporal trends, plotting on the *y* axis the estimated frequency of the *kdr* *L995F* allele in *Anopheles gambiae* complex species populations across the study health districts.

Regarding the *L995S* mutation, Banfora recorded the highest frequency in 2019 at 0.14 (95% CI 0.09–0.18), which decreased to 0.04 (95% CI 0.01–0.08; *P* < 0.001) in 2020, followed by a slight increase to 0.06 (95% CI 0.03–0.10; *P* = 0.03) in 2021. In Gaoua, the frequency of *L995S* remained consistently low throughout the period: 0.02 (95% CI 0.00–0.04) in 2019, 0.03 (95% CI 0.00–0.05; *P* = 0.71) in 2020 and 0.03 (95% CI 0.01–0.05; *P* = 0.509) in 2021. In Orodara, the frequency was 0.06 (95% CI 0.03–0.10) in 2019, followed by a non-significant reduction to 0.02 (95% CI 0.00–0.05; *P* = 0.114) in 2020, and it remained at 0.02 (95% CI 0.00–0.05; *P* = 0.06) in 2021.

The *kdr L995S* mutation was detected in all study health districts and across all *An. gambiae* complex species, although at relatively low frequencies. The highest frequency of the *kdr L995S* mutation, 0.13 (95% CI 0.07–0.29), was observed in the first year of the study in *An. gambiae* s.l. within the Banfora health district, on the basis of 206 samples tested including wild adult mosquitoes collected directly from the field and adults reared from wild-caught larvae. Overall, the *kdr L995S* mutation frequencies across all health districts remained low, with only minor fluctuations observed over the 3-year study period.

The proportion of individual mosquitoes carrying both *kdr* mutation alleles (*995F* and *995S*) was consistently below 30% across all years in each health district. In the first year, this proportion was 26.2% (51 out of 195) in Banfora, 2.9% (5 out of 170) in Gaoua and 12.5% (22 out of 176) in Orodara. In the second year, it was 6.9% (9 out of 130) in Banfora, 4.1% (7 out of 163) in Gaoua and 4.3% (7 out of 163) in Orodara. By the third year, the proportion was 6.7% (13 out of 194) in Banfora, 1.0% (2 out of 201) in Gaoua and 1.0% (2 out of 193) in Orodara.

## Discussion

The introduction of next-generation ITNs in Burkina Faso marks a critical turning point in the country’s vector control efforts, requiring robust monitoring of their impact on insecticide resistance. This study offers an in-depth, longitudinal assessment of phenotypic resistance to pyrethroids and the frequency of *kdr* mutations (*L995F* and *L995S*) in *An. gambiae* s.l. populations over a 3-year period in three health districts. Importantly, it incorporates two mosquito populations: adult females collected directly from the field and those reared from field-collected larvae in the insectary, providing a more nuanced understanding of resistance dynamics.

Despite the geographic proximity of the study districts, each exhibited distinct species compositions within the *An. gambiae* complex, shaped primarily by local ecological conditions. *An. gambiae* s.s. was predominant in Gaoua and Orodara, where mosquito breeding sites are largely temporary, rain-dependent pools, habitats that favour this species [48, 49]. Conversely, Banfora showed a co-dominance of *An. gambiae* s.s. and *An. coluzzii*, likely due to more permanent water sources such as a semi-permanent river and perennial ponds, which are the preferred breeding sites of *An. coluzzii* [49, 50]. These findings confirm that ecological heterogeneity plays a significant role in shaping vector population structure, sometimes outweighing the effects of vector control interventions.

Interestingly, significant differences in species composition were observed between field-collected wild adult mosquitoes (sampled continuously throughout the entire year) and adults reared from wild-caught larvae (collected only during the rainy season). These differences may, at least in part, be attributed to the timing of mosquito collection. Field-collected adults captured year-round sampling periods, thereby reflecting potential seasonal shifts in species dominance. Conversely, larval collections were limited to the rainy season, when suitable breeding habitats are abundant and may favour certain species over others. Previous studies have shown that *Anopheles* species exhibit marked seasonal variations in habitat preference and abundance, with rain-dependent temporary pools supporting higher larval densities and influencing species composition during the wet season [51]. Furthermore, seasonal changes have been linked to shifts in species dominance and microbial diversity within *Anopheles* populations, highlighting the dynamic nature of vector ecology throughout the year [52]. This discrepancy in temporal sampling windows likely contributed to the observed divergence in species composition, underlining the importance of accounting for seasonal variation when interpreting vector population dynamics.

The continued presence of *An. gambiae* s.l. across all study districts, including Gaoua, Orodara and Banfora, despite large-scale ITN deployment, likely reflects their ecological adaptability and resilience [53, 54]. The persistence of *An. gambiae* s.l. populations is not unexpected, especially in areas of high pyrethroids resistance. It is important to note that our study was conducted in areas of high-intensity pyrethroids resistance; therefore, the observed patterns and conclusions are particularly relevant to such contexts and should not be generalized to areas with low or moderate resistance levels. This highlights the importance of considering both ecological factors and resistance dynamics when interpreting species composition data. Mortality rates from WHO susceptibility bioassays were consistently below 45% across all study sites and years for both deltamethrin and alphacypermethrin, indicating high levels of resistance. Even when exposed to increased concentrations (5× and 10× the diagnostic doses), mosquito populations did not reach the 98% mortality threshold, confirming high-intensity resistance [55, 56]. These results are in line with previous national surveys highlighting widespread pyrethroid resistance in *An. gambiae* s.l. in Burkina Faso [13, 57, 58].

The pre-exposure to PBO**,** a synergist that inhibits cytochrome P450 enzymes, resulted in increased mortality, underscoring the contribution of metabolic resistance mechanisms to the observed phenotypes. However, mortality rates still failed to reach the WHO susceptibility threshold, suggesting that resistance is multifactorial, possibly involving additional mechanisms such as cuticular thickening or enhanced efflux pumps [59–61]. This partial restoration of susceptibility aligns with prior studies showing that PBO nets offer only transient or incomplete efficacy against highly resistant mosquito populations.

The *L995F* mutation was consistently more prevalent than *L995S* across all districts, years and mosquito populations. Its persistence in both adults that were field collected and adults that were reared from wild-caught larvae mosquitoes confirms its genetic stability in *An. gambiae* s.l. populations in this region [17, 18, 62]. In contrast, *L995S* mutation was detected at low frequencies and showed temporal fluctuations, particularly in field populations. This pattern is consistent with its relatively recent emergence in West African *An. gambiae* s.l. populations, including Burkina Faso [13, 63], where it remains much less common than in East Africa. Consequently, compound heterozygotes (*L995F/L995S*) were rare, which is more likely attributable to the low prevalence of the *L995S* allele rather than to any potential fitness cost associated with the co-occurrence of both mutations.

Moreover, recent studies have revealed the emergence and increasing frequency of alternative *kdr* haplotypes such as *V402L* and *I1527T*, in *An. gambiae* s.l. populations in Burkina Faso and neighbouring countries. These variants may also contribute to the decline of compound heterozygote by outcompeting the classical *L995* mutations [26, 64, 65].

One limitation of this study is the relatively small number of mosquitoes genotyped in some instances, which may have reduced the precision of allele frequency estimates and limited our ability to detect rare genotypes. Therefore, the results regarding *kdr* mutation frequencies should be interpreted with caution, and larger sample sizes would strengthen future analyses.

The trends in resistance were strongly associated with the type of ITN deployed in each district. In Gaoua, where Pyr-only nets were distributed, a consistent increase in *L995F* frequency and a decline in mortality rates in resistance assays were observed. This supports previous evidence that mono-insecticidal nets exert strong selection pressure for resistance alleles, especially when no synergist or secondary active ingredient is present [36, 66, 67]. In Banfora, the deployment of Pyr–CFR nets (combining a pyrethroid and chlorfenapyr) was associated with rising mortality rates in resistance assays and no increase in *kdr* mutation frequencies. Chlorfenapyr acts through mitochondrial disruption and bypasses the sodium channel target site, thereby circumventing *kdr*-mediated resistance and reducing selection pressure [67, 68]. This suggests Pyr–CFR nets may offer significant advantages in areas with intense *kdr*-based resistance.

In Orodara, the effects of Pyr–PBO nets were mixed. Mortality rates in resistance assays for deltamethrin initially decreased then rose again, whereas a steady decline in mortality rates was seen for alphacypermethrin, while *L995F* frequencies increased. These patterns suggest that PBO nets provided only partial and unstable improvements in efficacy, potentially owing to the persistence of multiple resistance mechanisms [32, 36, 69].

These findings demonstrate the complex interplay between ITN type, local ecology and resistance mechanisms. In this study context, which includes communities where agricultural insecticide use is common: Pyr-only ITNs continue to select strongly for *kdr* mutations, accelerating pyrethroid resistance in target vector; Pyr–CFR nets show promise in mitigating resistance and maintaining higher vector mortality; and Pyr–PBO nets offer an intermediate benefit, though their inconsistent performance underlines the need for more robust alternatives in areas of intense pyrethroids resistance.

Importantly, as the use of Pyr–CFR nets expands, proactive monitoring for emerging chlorfenapyr resistance, despite the current lack of extensive data, should be prioritized to ensure their long-term effectiveness. Consequently, integrated resistance management remains essential. This includes routine phenotypic and genotypic insecticide resistance surveillance, insecticide rotation, mosaic deployment of different ITN types and the use of complementary interventions like indoor residual spraying and larval source management [32, 43, 70]. The implementation of innovative tools as bioactive molecules from plants as essential oils would provide news insights in terms of vector control strategies [71–74].

## Conclusions

This study demonstrates that the type of ITN used can influence resistance outcomes in *An. gambiae* s.l. populations. Pyr-only nets were associated with intensified pyrethroid resistance and rising *L995F* mutation frequencies. Pyr–PBO nets provided partial but inconsistent improvements in mosquito mortality, suggesting limited effectiveness in high-resistance settings. In contrast, Pyr–CFR nets significantly improved mosquito mortality in resistance assays without selecting for *kdr* mutations, even in areas like western Burkina Faso where agricultural insecticide use and seasonal changes in mosquito populations are also likely to influence vector resistance dynamics. Altogether, the results underscore the need to prioritize the deployment of dual-active ingredient ITNs, rotating insecticides, and reinforcing resistance surveillance to maintain the effectiveness of malaria vector control in high-resistance settings.

## Supplementary Information

Additional file 1. Text S1. Allelic frequency calculation. Detailed description of the formulas used to estimate the allelic frequencies of the *kdr L995F* and *L995S* mutations in *Anopheles gambiae* s.l. populations. The formula accounts for homozygous and heterozygous genotypes and is applied across all sampled individuals to compute annual frequencies.

Additional file 2. Fig. S1–S2. Trends in mosquito mortality rates following exposure to increasing pyrethroid concentrations. The y-axis is displayed on a proportional scale (0 to 1). Fig. S1. Mortality rates of *Anopheles gambiae *s.l. populations following exposure to 0.25% alphacypermethrin and 0.25% deltamethrin across study years and health districts. Each point represents the average mortality rate for a given year and district, with vertical bars indicating 95% confidence intervals. Trends reflect changes in phenotypic resistance to intermediate concentrations of pyrethroids. Fig. S2. Mortality rates of *Anopheles gambiae* s.l. populations following exposure to 0.5% alphacypermethrin and 0.5% deltamethrin across study years and health districts. Higher insecticide concentrations were used to assess the intensity of resistance. As in Fig. S1, points represent average mortality rates with 95% confidence intervals.

Additional file 3. Table S1. Frequencies of *kdr L995F* and *L995S* alleles in adult *Anopheles gambiae *sensu lato populations collected from the field (wild-type) and reared from field-collected larvae (F0) across three study districts and years. This table presents the frequencies of *kdr-w (L995F)* and *kdr-e (L995S)* mutations, along with their associated 95% confidence intervals (CI), in three sibling species (*An. gambiae s.s.*, *An. coluzzii*, and *An. arabiensis*). Frequencies are shown by year (2019–2021), health district (Banfora, Gaoua, and Orodara), and mosquito origin (wild-type versus F0 laboratory-reared). Pyr–CFR area: District where Interceptor®G2 ITNs (pyrethroid + chlorfenapyr) were deployed (Banfora); Pyr-only area: District where Interceptor® G1 ITNs (pyrethroid-only) were deployed (Gaoua); Pyr–PBO area: District where PermaNet®3.0 ITNs (pyrethroid + PBO) were deployed (Orodara). N: number of individuals analyzed per combination of district, species, and year. freq(F): frequency of the *L995F* allele (*kdr-w*); freq(S): frequency of the *L995S* allele (*kdr-e*); 95% CI: 95% confidence interval of the estimated allele frequency. “Wild-type” refers to adult mosquitoes collected directly in the field; “F0” refers to adult mosquitoes reared in the laboratory from larvae collected in the field.

## Data Availability

Data supporting the main conclusions of this study are included in the manuscript.

## Abbreviations

CDC-LT: United State Center for Diseases Control and Prevention Light Trap
ITN: Insecticide-treated bed net
IRS: Indoor residual spraying
IR: Insecticide resistance
VGSC: Voltage-gated sodium channel
CNRFP: Centre National de Recherche et de Formation sur le Paludisme
WHO: World Health Organization
VCRU: Vector Control; Research Unit at University Sains Malaysia
Dual-AI: Dual active ingredients
HLC: Human landing catches
DC: Diagnostic concentration
GLM: Generalized linear model
*kdr*: Knockdown resistance
NMCP: National Malaria Control Program
PBO: Piperonyl butoxide
PCR: Polymerase chain reaction
95% CI: 95% confidence interval
Pyr–CFR: Pyrethroid + chlorfenapyr
Pyr-only: Pyrethroid-only
Pyr–PBO: Pyrethroid + piperonyl butoxide
WT: Wild type
