# Supplementary material for: Differential effects of dual and synergist-based insecticide-treated bed nets on pyrethroid resistance and L995F/S knockdown resistance mutation dynamics in Anopheles gambiae s.l. populations in south-western Burkina Faso
Source: Parasit Vectors. 2025 Dec 20;19:46. doi: 10.1186/s13071-025-07190-3 (PMC12838508; doi:10.1186/s13071-025-07190-3)
Supplement: Supplementary file 1 — Additional file 1. Text S1. Allelic frequency calculation. Detailed description of the formulas used to estimate the allelic frequencies of the kdr L995F and L995S mutations in Anopheles gambiae s.l. populations. The formula accounts for homozygous and heterozygous genotypes and is applied across all sampled individuals to compute annual frequencies. [file 13071_2025_7190_MOESM1_ESM.docx]

**Text S1. Allelic frequency calculation**

The frequencies of *kdr* mutations *L995F* and *L995S* were calculated as follows:

- The frequency of the ***L995F*** allele, denoted as freq(F), was calculated using the formula:
  **freq(F) = (2 × FF + LF + FS) / 2n**
- The frequency of the ***L995S*** allele, denoted as freq(S), was calculated using the formula:
  **freq(S) = (2 × SS + LS + FS) / 2n**

Where:

- **FF** and **SS** represent the number of mosquitoes homozygous for the *L995F* and *L995S* mutations, respectively.
- **LF**, **LS**, and **FS** represent heterozygous individuals carrying combinations of both alleles.
- **n** is the total number of mosquitoes successfully genotyped.

These formulas ensure that both homozygous and heterozygous genotypes are considered in estimating the overall allelic frequency in the population.
