# Supplementary material for: Differential effects of dual and synergist-based insecticide-treated bed nets on pyrethroid resistance and L995F/S knockdown resistance mutation dynamics in Anopheles gambiae s.l. populations in south-western Burkina Faso
Source: Parasit Vectors. 2025 Dec 20;19:46. doi: 10.1186/s13071-025-07190-3 (PMC12838508; doi:10.1186/s13071-025-07190-3)
Supplement: Supplementary file 2 — Additional file 2. Fig. S1–S2. Trends in mosquito mortality rates following exposure to increasing pyrethroid concentrations. The y-axis is displayed on a proportional scale (0 to 1). Fig. S1. Mortality rates of Anopheles gambiae s.l. populations following exposure to 0.25% alphacypermethrin and 0.25% deltamethrin across study years and health districts. Each point represents the average mortality rate for a given year and district, with vertical bars indicating 95% confidence intervals. Trends reflect changes in phenotypic resistance to intermediate concentrations of pyrethroids. Fig. S2. Mortality rates of Anopheles gambiae s.l. populations following exposure to 0.5% alphacypermethrin and 0.5% deltamethrin across study years and health districts. Higher insecticide concentrations were used to assess the intensity of resistance. As in Fig. S1, points represent average mortality rates with 95% confidence intervals. [file 13071_2025_7190_MOESM2_ESM.docx]

**Fig. S1–S2. Trends in mosquito mortality rates following exposure to increasing pyrethroid concentrations. Fig. S1. Mortality rates of *Anopheles gambiae* s.l. populations following exposure to 0.25% alphacypermethrin and 0.25% deltamethrin across study years and health districts.** Each point represents the average mortality rate for a given year and district, with vertical bars indicating 95% confidence intervals. Trends reflect changes in phenotypic resistance to intermediate concentrations of pyrethroids. **Fig. S2. Mortality rates of *Anopheles gambiae* s.l. populations following exposure to 0.5% alphacypermethrin and 0.5% deltamethrin across study years and health districts.** Higher insecticide concentrations were used to assess the intensity of resistance. As in **Fig. S1**, points represent average mortality rates with 95% confidence intervals.

***Fig. S1. Mortality rates of Anopheles gambiae s.l. populations following exposure to 0.25% alphacypermethrin and 0.25% deltamethrin across study years and health districts.****
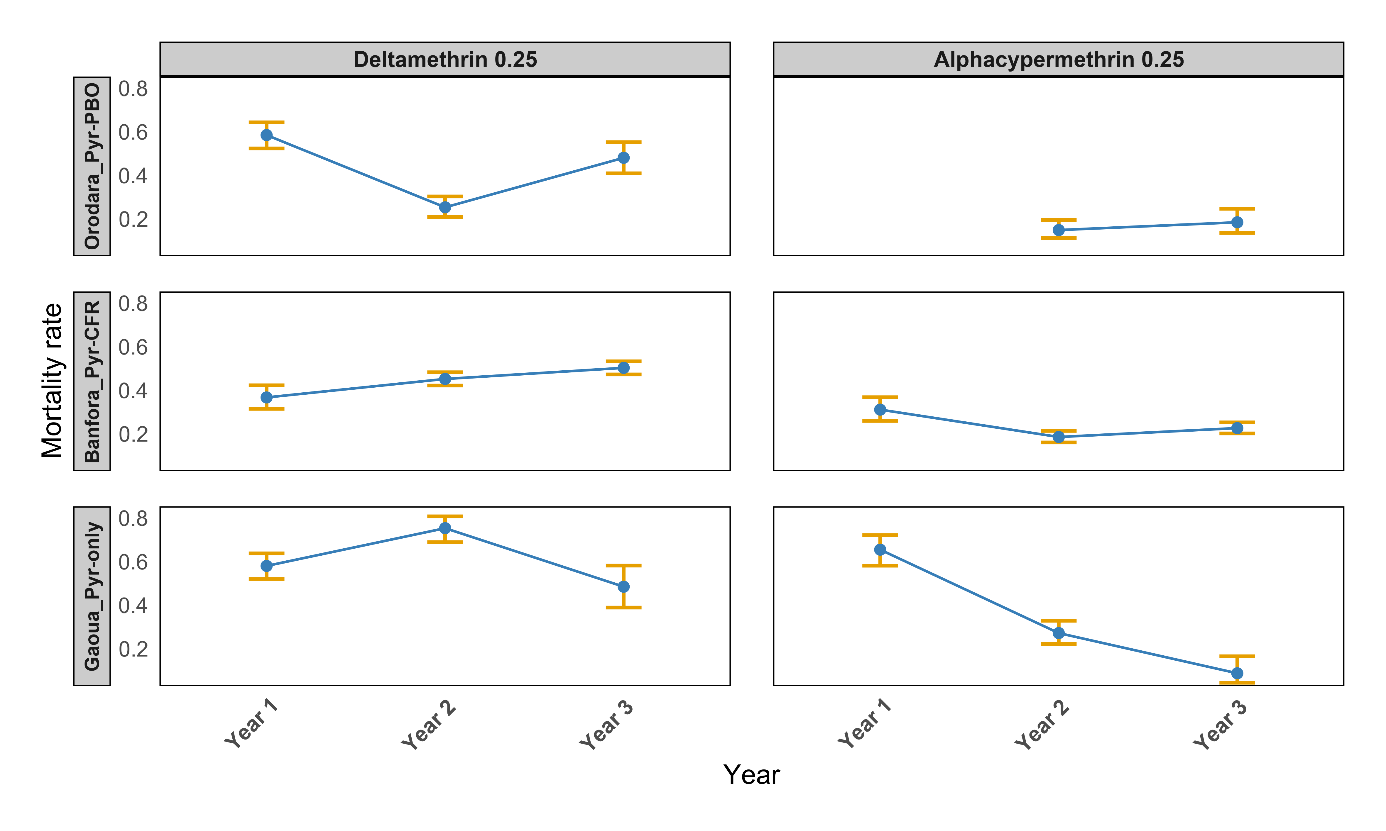
*

***Fig. S2. Mortality rates of Anopheles gambiae s.l. populations following exposure to 0.5% alphacypermethrin and 0.5% deltamethrin across study years and health districts.****
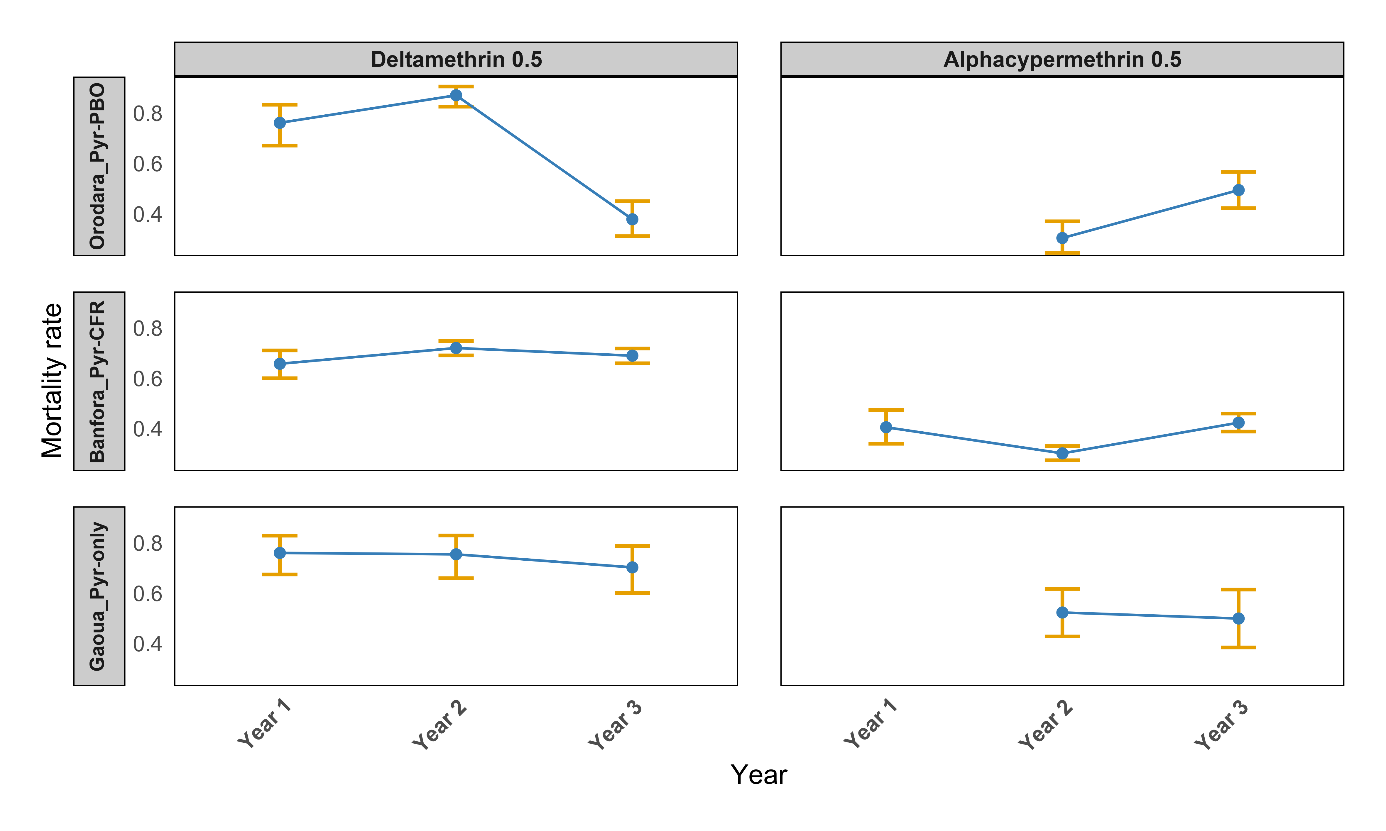
*
