# Supplementary material for: Differential effects of dual and synergist-based insecticide-treated bed nets on pyrethroid resistance and L995F/S knockdown resistance mutation dynamics in Anopheles gambiae s.l. populations in south-western Burkina Faso
Source: Parasit Vectors. 2025 Dec 20;19:46. doi: 10.1186/s13071-025-07190-3 (PMC12838508; doi:10.1186/s13071-025-07190-3)
Supplement: Supplementary file 3 — Additional file 3. Table S1. Frequencies of kdr L995F and L995S alleles in adult Anopheles gambiae sensu lato populations collected from the field (wild-type) and reared from field-collected larvae (F0) across three study districts and years. This table presents the frequencies of kdr-w (L995F) and kdr-e (L995S) mutations, along with their associated 95% confidence intervals (CI), in three sibling species (An. gambiae s.s., An. coluzzii, and An. arabiensis). Frequencies are shown by year (2019–2021), health district (Banfora, Gaoua, and Orodara), and mosquito origin (wild-type versus F0 laboratory-reared). Pyr–CFR area: District where Interceptor®G2 ITNs (pyrethroid + chlorfenapyr) were deployed (Banfora); Pyr-only area: District where Interceptor® G1 ITNs (pyrethroid-only) were deployed (Gaoua); Pyr–PBO area: District where PermaNet®3.0 ITNs (pyrethroid + PBO) were deployed (Orodara). N: number of individuals analyzed per combination of district, species, and year. freq(F): frequency of the L995F allele (kdr-w); freq(S): frequency of the L995S allele (kdr-e); 95% CI: 95% confidence interval of the estimated allele frequency. “Wild-type” refers to adult mosquitoes collected directly in the field; “F0” refers to adult mosquitoes reared in the laboratory from larvae collected in the field. [file 13071_2025_7190_MOESM3_ESM.docx]

Table S1 Frequencies of kdr L995F and L995S alleles in adult *Anopheles gambiae sensu lato* populations collected directly from the field (WT) and in adults reared from wild-caught larvae (F0) across three study districts and years.

| Collection year | Tested number/Frequency |  | Banfora (Pyr-CFR area) | | |  | Gaoua (Pyr-only area) | | |  | Orodara (Pyr-PBO area) | | |
| --- | --- | --- | --- | --- | --- | --- | --- | --- | --- | --- | --- | --- | --- |
|  |  |  | WT | F0 | WT+F0 |  | WT | F0 | WT+F0 |  | WT | F0 | WT+F0 |
| Year 1 | *An. gambiae* s.s. | | | | | | | | | | | | |
|  | N |  | 73 | 11 | 84 |  | 148 | 15 | 163 |  | 129 | 23 | 152 |
|  | Freq.F [95%CI] |  | 0.86 [0.78–0.94] | 0.90 [0.7–1.08] | 0.87 [0.77–0.96] |  | 0.55 [0.47–0.63] | 0.97 [0.8 –1.06] | 0.59 [0.43– 0.67] |  | 0.67 [0.59– 0.76] | 0.89 [0.76–1.02] | 0.70 [0.62–0.80] |
|  | Freq.S [95%CI] |  | 0.12 [0.05–0.20] | 0.0 | 0.10 [0.04–0.17] |  | 0.01 [0.0–0.02] | 0.0 | 0,.01 [0.0– 0.02] |  | 0.02 [0.0–0.04] | 0.09 [0.0–0.20] | 0.03 [0.0–0.06] |
|  | *An. coluzzii* | | | | | | | | | | | | |
|  | N |  | 86 | 32 | 118 |  | 10 | 0 | 10 |  | 23 | 3 | 26 |
|  | Freq.F [95%CI] |  | 0.76 [0.67–0.85] | 0.75 [0.60–0.90] | 0.76 [0.65–0.86] |  | 0.5 [0.19–0.80] | NA | 0.5 [0.19– 0.8] |  | 0.5 [0.30–0.70] | 0.5 [0.07–1.07] | 0.5 [0.27–0.74] |
|  | Freq.S [95%CI] |  | 0.22 [0.13–0.31] | 0.0 | 0.16 [0.09–0.22] |  | 0.2 [0.0–0.45] | NA | 0.2 [0.0– 0.45] |  | 0.30 [0.12–0.49] | 0.0 | 0.27 [0.11–0.43] |
|  | *An. arabiensis* | | | | | | | | | | | | |
|  | N |  | 1 | 3 | 4 |  | 1 | 5 | 6 |  | 0 | 0 | 0 |
|  | Freq.F [95%CI] |  | 0.5 [0.0–1.48] | 0.83 [0.41–1.25] | 0.74 [0.31–1.31] |  | 0.5 [0.0–1.48] | 0.7 [0.30–1.10] | 0.67 [0.25– 1.16] |  | NA | NA | NA |
|  | Freq.S [95%CI] |  | 0.5 [0.0–1.48] | 0.0 | 0.12 [0.0–0.37] |  | 0.0 | 0.0 | 0.0 |  | NA | NA | NA |
|  | *An. gambiae* s.l. | | | | | | | | | | | | |
|  | N |  | 160 | 46 | 206 |  | 159 | 20 | 179 |  | 152 | 26 | 178 |
|  | Freq.F [95%CI] |  | 0.80 [0.72–1.23] | 0.79 [0.61–1.56] | 0.80 [0.75–0.86] |  | 0.55 [0.45–0.67] | 0.90 [0.66–1.24] | 0.58 [0.51–0.66] |  | 0.64 [0.55–0.81] | 0.84 [0.68–1.09] | 0.68 [0.61–0.75] |
|  | Freq.S [95%CI] |  | 0.18 [0.09–0.37] | 0 | 0.13 [0.07–0.29] |  | 0.02 [0.0–0.05] | 0.18 [0.08–0.31] | 0.02 [0.0–0.05] |  | 0.06 [0.02–0.12] | 0.08 [0.0–0.19] | 0,07 [0.02–0.12] |
| Year 2 | *An. gambiae* s.s. | | | | | | | | | | | | |
|  | N |  | 33 | 24 | 57 |  | 72 | 42 | 114 |  | 77 | 71 | 148 |
|  | Freq.F [95%CI] |  | 0.95 [0.88–1.02] | 0.94 [0.84–1.03] | 0.95 [0.86–1.02] |  | 0.97 [0.92–1.01] | 0.94 [0.90–1.01] | 0.96 [0.87–1.01] |  | 0.99 [0.97–1.01] | 0.96 [0.91–1.00] | 0.97 [0.94–1.00] |
|  | Freq.S [95%CI] |  | 0.03 [0.0–0.09] | 0.04 [0.0–0.12] | 0.03 [0.0–0.10] |  | 0.02 [0.0–0.05] | 0.02 [0.0–0.05] | 0.01 [0.0–0.04] |  | 0.01 [0.0–0.02] | 0.01 [0.0–0.04] | 0.01 [0.0–0.03] |
|  | *An. coluzzii* | | | | | | | | | | | | |
|  | N |  | 48 | 22 | 70 |  | 10 | 16 | 26 |  | 11 | 0 | 11 |
|  | Freq.F [95%CI] |  | 0.60 [0.47–0.74] | 0.68 [0.49–0.88] | 0.63 [0.48–0.78] |  | 0.5 [0.19–0.81] | 0.63 [0.39–0.86] | 0.58 [0.31–0.84] |  | 0.64 [0.35–0.92] | NA | 0.64 [0.35–0.92] |
|  | Freq.S [95%CI] |  | 0.05 [0.0–0.11] | 0.0 | 0.03 [0.0–0.08] |  | 0.2 [0.0–0.45] | 0.0 | 0.08 [0.0–0.17] |  | 0.18 [0.0–0.41] | NA | 0.18 [0.0–0.41] |
|  | *An. arabiensis* | | | | | | | | | | | | |
|  | N |  | 0 | 5 | 5 |  | 2 | 16 | 18 |  | 1 | 4 | 5 |
|  | Freq.F [95%CI] |  | NA | 0.5 [0.06–0.94] | 0.5 [0.06–0.94] |  | 0.25 [0.0–0.85] | 0.59 [0.35–0.83] | 0.55 [0.31–0.83] |  | 0.5 [0.0–1.48] | 0.75 [0.33–1.17] | 0.70 [0.26–1.23] |
|  | Freq.S [95%CI] |  | NA | 0.20 [0.0– 0.55] | 0.20 [0.0– 0.55] |  | 0.0 | 0.0 | 0.0 |  | 0.0 | 0.13 [0.0–0.45] | 0.10 [0.0–0.36] |
|  | *An. gambiae s.l.* | | | | | | | | | | | | |
|  | N |  | 81 | 51 | 132 |  | 84 | 74 | 158 |  | 89 | 75 | 164 |
|  | Freq.F [95%CI] |  | 0.74 [0.64–1.21] | 0.78 [0.61–1.3] | 0.76 [0.69–0.83] |  | 0.90 [0.81–1.06] | 0.80 [0.67–1.20] | 0.85 [0.79–0.90] |  | 0.94 [0.88–1.08] | 0.95 [0.88–1.04] | 0.95 [0.91–0.98] |
|  | Freq.S [95%CI] |  | 0.04 [0.0– 0.14] | 0.04 [0.0– 0.15] | 0.04 [0.0– 0.15] |  | 0.04 [0.0– 0.10] | 0.01 [0.0– 0.04] | 0.02 [0.0– 0.07] |  | 0.03 [0.0–0.07] | 0.02 [0.0–0.06] | 0.02 [0.0–0.07] |
| Year 3 | *An. gambiae* s.s. | | | | | | | | | | | | |
|  | N |  | 52 | 31 | 83 |  | 120 | 16 | 136 |  | 104 | 49 | 153 |
|  | Freq.F [95%CI] |  | 0.95 [0.89–1.01] | 1.0 | 0.97 [0.93–1.01] |  | 0.92 [0.87–0.97] | 0.99 [0.96–1.02] | 0.93 [0.88–0.97] |  | 0.89 [0.83–0.95] | 0.92 [0.84–0.99] | 0.90 [0.83–0.96] |
|  | Freq.S [95%CI] |  | 0.01 [0.0–0.04] | 0.0 | 0.01 [0.0–0.03] |  | 0.03 [0.0–0.07] | 0.0 | 0.03 [0.0–0.06] |  | 0.03 [0.0–0.07] | 0.0 | 0.02 [0.0–0.05] |
|  | *An. coluzzii* | | | | | | | | | | | | |
|  | N |  | 75 | 13 | 88 |  | 7 | 15 | 22 |  | 32 | 4 | 36 |
|  | Freq.F [95%CI] |  | 0.58 [0.47–0.69] | 0.59 [0.42–0.77] | 0.58 [0.46–0.70] |  | 0.86 [0.60–1.12] | 0.5 [0.25–0.75] | 0.61 [0.36–0.87] |  | 0.88 [0.76–0.99] | 0.88 [0.55–1.20] | 0.88 [0.74–1.01] |
|  | Freq.S [95%CI] |  | 0.12 [0.05–0.19] | 0.0 | 0.10 [0.04–0.16] |  | 0.07 [0.0–0.26] | 0.0 | 0.02 [0.0–0.28] |  | 0.03 [0.0–0.09] | 0.0 | 0.03 [0.0–0.08] |
|  | *An. arabiensis* | | | | | | | | | | | | |
|  | N |  | 4 | 6 | 10 |  | 6 | 7 | 13 |  | 0 | 1 | 1 |
|  | Freq.F [95%CI] |  | 0.13 [0.0–0.45] | 0.58 [0.19–0.38] | 0.4 [0.11–0.51] |  | 0.67 [0.29–1.04] | 0.43 [0.06–0.80] | 0.54 [0.17–0.91] |  | NA | 0.5 [0.0–1.48] | 0.5 [0.0–1.48] |
|  | Freq.S [95%CI] |  | 0.5 [0.01– 0.99] | 0.0 | 0.02 [0.0–0.40] |  | 0.25 [0.0–0.60] | 0.0 | 0.12 [0.0–0.28] |  | NA | 0.0 | 0.0 |
|  | *An. gambiae* s.l. | | | | | | | | | | | | |
|  | N |  | 131 | 50 | 181 |  | 133 | 38 | 171 |  | 136 | 54 | 190 |
|  | Freq.F [95%CI] |  | 0.71 [0.62–1.16] | 0.84 [0.75–1.07] | 0.71 [0.64–0.78] |  | 0.91 [0.83–1.03] | 0.69 [0.51–1.23] | 0.88 [0.84–0.93] |  | 0.89 [0.81–1.09] | 0.91 [0.80–1.06] | 0.89 [0.85–0.94] |
|  | Freq.S [95%CI] |  | 0.09 [0.03– 0.22] | 0.0 | 0.05 [0.02– 1.16] |  | 0.04 [0.0– 0.11] | 0.0 | 0.03 [0.0– 0.12] |  | 0.03 [0.0– 0.08] | 0.0 | 0.02 [0.0– 0.06] |
